# Supplementary material for: Complexome profiling of the Chlamydomonas psb28 mutant reveals TEF5 as an early PSII assembly factor
Source: Plant Cell. 2025 Mar 17;37(6):koaf055. doi: 10.1093/plcell/koaf055 (PMC12164590; doi:10.1093/plcell/koaf055)
Supplement: koaf055_Supplementary_Data [file koaf055_supplementary_data.zip › psb28_interactive.zip!/plots/intensity/qualityPlots/peptides/PST1_Cre07.g331550.t1.2.html]

### Cre07.g331550.t1.2

Trivial name: PST1  
SeqCoverage [median]: 13.60  
Mass: 44.13 kDa  
  
 

| Sequence | MissedCleavage | Mass [Da] | wtIntensity | muIntensity | Score |
| --- | --- | --- | --- | --- | --- |
| ASIYNAMPIEGVQK | 0 | 1519.77 | 0.00 | 431.89 | 66.60 |
| LGGLDAMQELNR | 0 | 1315.66 | 0.00 | 574.29 | 80.98 |
| VLVSDMSSNFISKPIDVAK | 1 | 2049.08 | 0.00 | 1285.91 | 41.86 |
| YGIIYAGAQK | 0 | 1082.58 | 0.00 | 533.03 | 79.12 |
|  |  |  | 0.0 | 2825.1 |  |
